# Serum-Free Cultures: Could They Be a Future Direction to Improve Neuronal Differentiation of Mesenchymal Stromal Cells?

**DOI:** 10.3390/ijms23126391

**Published:** 2022-06-07

**Authors:** Giovanni Schepici, Agnese Gugliandolo, Emanuela Mazzon

**Affiliations:** IRCCS Centro Neurolesi “Bonino-Pulejo”, Via Provinciale Palermo, Contrada Casazza, 98124 Messina, Italy; giovanni.schepici@irccsme.it (G.S.); agnese.gugliandolo@irccsme.it (A.G.)

**Keywords:** mesenchymal stem/stromal cells, serum-free media, cell proliferation, neuronal differentiation, in vitro studies

## Abstract

Mesenchymal stem/stromal cells (MSCs) are undifferentiated cells with multilinear potential, known for their immunomodulatory and regenerative properties. Although the scientific community is working to improve their application, concerns limit their use to repair tissues following neurological damage. One of these obstacles is represented by the use of culture media supplemented with fetal bovine serum (FBS), which, due to its xenogenic nature and the risk of contamination, has increased scientific, ethical and safety problems. Therefore, the use of serum-free media could improve MSC culture methods, avoiding infectious and immunogenic transmission problems as well as MSC bioprocesses, without the use of animal components. The purpose of our review is to provide an overview of experimental studies that demonstrate that serum-free cultures, along with the supplementation of growth factors or chemicals, can lead to a more defined and controlled environment, enhancing the proliferation and neuronal differentiation of MSCs.

## 1. Introduction

Mesenchymal stromal cells (MSCs) are known for their high self-renewal capacity, multilinear differentiation potential and immunomodulatory properties, making them an interesting tool for research and clinical applications. Although MSC properties may depend on multiple parameters, including the tissue source, medium composition and method of isolation, MSC identification requires several biomarkers [1]. According to the International Society for Cellular Therapy (ISCT), MSC characterization needs to satisfy minimum criteria, including: their plastic adherence under standard culture conditions; the ability to differentiate in vitro towards chondroblasts, adipocytes and osteoblasts; the lack of hematopoietic markers CD34, CD45 and CD14, CD79α or CD19 and HLA-DR as well as the expression of surface molecules CD90, CD105 and CD73 [2]. MSCs were first identified and isolated from bone marrow (BM), but the invasive harvesting process makes the use of BM-derived MSCs (BM-MSCs) limited. Consequently, it is preferred to use MSCs from other tissues compared to BM-MSCs [2,3]. In this regard, alternative sources of human MSCs (hMSCs), including adipose tissue (AT), umbilical cord (UC), dental tissues, birth-derived tissues, etc., were found. The high yield and easy collection procedure make AT an excellent source of MSCs for use in tissue engineering [4]. It was also reported that tooth-derived MSCs originate from the neural crest and can be used for neurological applications [5].

Among tooth-derived MSCs, particularly promising are the dental pulp-derived MSCs (DP-MSCs), which show the advantage of being easily accessible for extraction from periodontal tissues. Moreover, DP-MSCs demonstrate the ability to differentiate into odontoblasts, osteoblasts, chondrocytes, adipocytes and neurons [6].

DP-MSCs can express specific neuronal markers, including nestin, glial fibrillary acidic protein (GFAP) and S100ß, as well as prion protein, highly expressed in neuronal differentiation. Several studies reported the DP-MSC property as a potential application for neural induction and regeneration [7,8,9]. Indeed, it was demonstrated that DP-MSCs can differentiate into dopaminergic neuron-like cells or neuronal-like cells [10]. Moreover, it was observed that DP-MSCs cultivated in neuronal inductive media can release trophic factors, including the brain-derived neurotrophic factor (BDNF), GDNF and nerve growth factor (NGF), to induce an increased proliferation rate of Schwann cells and neurite growth [11].

Thanks to their neurogenic and angiogenic potential, DP-MSCs can be investigated in the treatment of nervous system diseases [12].

Overall, it was suggested that DP-MSCs could be a useful cellular model to investigate neurodegenerative diseases, including Alzheimer’s disease, Parkinson’s disease and Huntington’s disease [13].

Moreover, perinatal tissues, such as chorion, amnion and Wharton’s jelly (WJ), are also important sources of MSCs [14].

Although MSCs can differentiate toward various cell populations thanks to their multilinear differentiation potential, their application in a clinical setting requires overcoming several challenges [15]. In this regard, it is essential to develop standardized procedures that allow for a consistent MSC characterization. The process of isolation and collection from the donor as well as the culture conditions could influence the cell population propagated in vitro and also the number of cells obtained. Since MSC clinical applications require a number of cells higher than that isolated from the tissue itself, it is necessary to identify optimal culture conditions, taking into consideration different parameters, including pH, temperature, medium compositions, cell seeding density, oxygen (O_2_) and carbon dioxide (CO_2_) concentrations. Considering the complexity of culture media, a qualitative and quantitative improvement of culture media remains a goal for researchers [16].

Consequently, developing culture conditions such as serum-free media (SFM) could be useful for optimizing culture media and increasing quantitative and qualitative yields [17].

The aim of this review is to summarize the experimental studies that highlight how applications of SFM, also with the supplementation of growth factors or chemicals, can improve MSC neuronal differentiation properties, to favor their use as a potential treatment for a wide range of debilitating diseases, including neurodegenerative ones.

## 2. Serum in MSC Cultures

The regenerative potential of MSCs has been known for decades, meaning they could be a promising treatment for disabling diseases. To stimulate growth and proliferation, the integration of serum is necessary. Serum is a complex mixture consisting of a large number of constituents, including biomolecules with biological activity that act on cell growth. The main functions of serum in culture media are to provide hormonal factors capable of stimulating proliferation, growth and cell differentiation, transport proteins, components of extracellular matrix and factors necessary both to maintain pH and with detoxifying action. To date, specific media for different cell types can be supplemented with a variety of protein sources, including FBS, human serum, bovine serum albumin (BSA) and human albumin. FBS contains a high concentration of growth factors, which are nutritional and chemical compounds necessary for the maintenance and expansion of MSCs [18]. Therefore, the high risk of contamination, immunizing effects as well as controversies on the therapeutic outcome, have led researchers to study alternative compounds to FBS.

## 3. Alternatives to Animal Serum in MSC Cultures

Several alternatives to animal serum to improve the proliferation and differentiation capacity of MSCs; such as culture medium supplemented with human-derived serum, including autologous or allogeneic human serum, umbilical cord blood serum (UCBS) and human platelet lysates (hPL); were investigated by researchers [19,20].

### 3.1. Human Serum in MSC Cultures

One of the advantages in the use of a human-blood-derived compared to animal serum can be the absence of risks related to FBS constituents for hMSC expansion culture [16,21], while one of the limitations related to the use of blood-derived constituents could be the possibility of contamination by pathogens not detected by the screening performed on donors. Therefore, filtration through 0.2 μm porous membranes could be useful for the elimination of some bacteria, but not viruses smaller than 0.1 μm in size. Additionally, procedures such as photochemical or ultraviolet-A light treatments have proven to be effective in improving quality standards [22]. Moreover, a further disadvantage could be the nonhomogenous expansion of hMSCs induced by the high variability among human blood samples [19].

Several studies reported the effects of human serum on MSC cultures compared to animal serum. In the past few years, a greater proliferative capacity of MSCs cultivated with human serum compared to MSCs cultivated with FBS has been demonstrated [23].

In this regard, it has been shown that the use of human autologous serum (HAS) at 10% in the absence of growth factors and cytokines induced effects similar to FBS 10% on the stimulation and growth of hMSCs [24,25,26].

Additionally, a shorter doubling time of hMSCs cultivated with HAS compared to FBS was highlighted. Moreover, the gene expression analysis of hMSCs supplemented with HAS demonstrated a reduction in the differentiation capacity and an increase in the proliferation of hMSCs, probably due to the involvement of factors, including Angiopoietin-like 4 (Angptl4), which acts as a survival factor to apoptosis. Contrarily, hMSCs cultivated with FBS have shown an upregulation of genes involved in both the differentiation and cell cycle. Overall, a rapid expansion in HAS-cultivated hMSCs in the absence of growth factors was reported. Noteworthy is that HAS-supplemented hMSCs were transcriptionally more stable over culture time, but less differentiated compared to FBS-cultivated hMSCs [24].

In contrast, it was reported that the use of allogeneic serum induced a longer time of hMSC adhesion and proliferation compared to the FCS-cultivated hMSCs. This could probably be explained by the presence of some growth factors in the allogeneic serum, which led to an inhibitory effect on the expansion and survival of hMSCs compared to the FCS group [27].

The results discussed thus far are presented in Table 1.

### 3.2. Human Umbilical Cord Blood Serum in MSC Cultures

For decades, it has been known that umbilical cord blood is a rich source of growth factors capable of supporting the proliferation, growth and differentiation of stem cells residing in fetal blood [28]. In this regard, human umbilical cord blood serum (hUCBS) could be useful to support the in vitro expansion of MSCs. Although there are currently no comprehensive studies regarding this, the presence of a greater number of growth factors in allogeneic human placenta or cord blood serum appears to improve the expansion efficiency of hMSCs compared to FBS-treated cells [29,30].

One of the reasons why hUCBS may be suitable for hMSC cultures could probably be the presence of serum albumin and transferrin [31]. Human serum albumin, through its interaction with small molecules, can regulate processes involved in growth and cell proliferation, including the distribution of cellular components, intra- and extracellular transport of molecules and apoptosis [19]. While transferrin acting as an iron transporter can directly modulate the cell cycle, a higher transferrin level is required for cell-proliferating compared to cell-differentiating ones [32,33,34].

Moreover, it was demonstrated that media integrated with hUCBS promotes the growth, proliferation and differentiation of human BMSCs compared to FCS-cultivated ones. The presence of growth factors could explain the efficacy of the fetal or newborn serum on the grown, proliferation and differentiation of MSCs. Therefore, compared to fetal calf/bovine serum, the use of hUCBS does not involve the problem of xenoproteins in a transplant and, thus, could be a better supplement [28].

Additionally, hBM-MSCs cultivated in 10% hUCBS-supplemented media showed exponential growth with a shorter doubling time than those in 10% FBS-supplemented media. Noteworthy is that hBM-MSCs cultivated with hUCBS supplemented showed a shift from adipogenic to osteogenic differentiation potential, probably due to some unknown action factors present in UCBS. Unlike the FBS-supplemented culture, hMSCs cultivated with hUCBS demonstrated a high level of osteopontin, osteocalcin and alkaline phosphatase during the proliferative phase of growth. Contrarily, in cultures supplemented with FBS, these proteins were expressed only during the osteogenic differentiation of hMSCs. Consequently, the variability in the differentiation patterns induced by hUCBS-grown hMSCs led to tests examining the efficacy of hUCBS as a supplement for differentiation towards other lineages, such as neuronal ones [29].

Overall, hUCBS is an abundantly available allogeneic serum, which also exhibits the advantage of being free of xenogenic contaminants. Additionally, hUCBS can be easily isolated, and is considered safe and stable compared to cultures supplemented with animal serum. However, variability from batch to batch and the presence of any pathogens that were not detected during routine screening could represent disadvantages for its therapeutic application [19].

The results discussed thus far are presented in Table 2.

### 3.3. Human Platelet Derivatives in MSC Cultures

Recent studies have investigated platelet lysate formulation-like alternatives to animals serum to support the growth, proliferation and differentiation of hMSCs, as well as to replace the use of FBS [35].

Platelet lysate is obtained from platelet-rich plasma, which is isolated from uncoagulated blood. The preparation of platelet lysate can take place either by mechanical breaking or by freeze/thaw cycles to destroy the platelets through the formation of ice crystals. Additionally, platelet lysate can be obtained by adding calcium, collagen or thrombin to trigger the coagulatory cascade. The fibrinogen coagulated by centrifugation and microfiltration is separated from the liquid suspension containing the platelet lysate [36].

Platelet activation and the subsequent release of growth factors, including platelet-derived growth factors (PDGFs) -AA, -AB and -BB, transforming growth factors (TGFs) -β1 and β2, epidermal growth factor (EGF), basic fibroblast growth factor (bFGF), BDNF and hepatocyte growth factor (HGF), along with a variety of cytokines interleukin (IL)-1α, IL-7, IL-8 and interferon (IFN) -γ, as well as high concentrations of cell adhesion molecules, attachment factors (fibronectin and vitronectin), coagulation factors and other biomolecules could be useful in replacing animal serum in hMSC cultures [37,38].

It was reported that the presence of PDGF, TGF-β and bFGF in hPL can influence cell morphogenesis, proliferation and differentiation [39,40,41,42]. Therefore, to expand and differentiate MSCs towards a specific lineage, the choice of growth factors is fundamental. Although the presence of PDGF-AB/BB, TGF-β1 and bFGF seems necessary for an optimal expansion, the results through their use alone are insufficient. Indeed, it was demonstrated that for the optimal expansion and full biological activity of MSCs, in addition to the essential components PDGF-BB, TGF-β1 and bFGF, other constituents present in hPL are necessary [43].

The presence of platelet-derived growth factors prompted researchers to perform studies on hMSCs. In this regard, it was shown that hPL supplementation improved the proliferation of human endothelial cells and hMSCs, probably thanks to the presence of platelet-derived growth factors. In particular, it was also demonstrated that human PDGF uniquely promoted the migration and proliferation of both hMSCs and endothelial cells [20].

TGF-β is known to influence MSCs towards the chondrogenic lineage. Indeed, it was demonstrated that TGF-β induced the chondrocytes proliferation as well as the deposition of an extracellular matrix (ECM) and specific cartilage molecules [44].

Similarly, it was shown in vitro that TGF-β supplementation promoted proliferation and differentiation towards a chondrogenic lineage of MSCs [45].

Noteworthy is that the combined effect of TGF-β3 and bone morphogenic protein (BMP)-2 belonging to the TGF-β family led to a greater increase in proliferation and differentiation towards a chondrogenic lineage of MSCs. Therefore, the combined use of growth factors could potentially be useful for regenerative medicine [46].

Basic growth factors such as EGF and FGF can promote the expansion and survival of MSCs. In fact, it was demonstrated that EGF improved cell proliferation and survival when bound with MSCs on a biomaterial surface. EGF enhanced the contact of MSCs with a synthetic polymer in an in vivo model of inflammation. Therefore, EGF-modified polymers could be used for the development of MSC scaffolds useful for the treatment of lesions [47].

Several growth factors also improved MSC survival. In this regard, it was demonstrated that the transplantation of MSCs with NGF and BDNF induced a significant increase in transplanted cells in a mouse model of a brain injury compared to MSCs transplanted without a growth factor [48].

Although HGF had a limited effect on cell proliferation, it was also observed that this growth factor led to an increase in MSC survival [49].

Several studies have demonstrated that hPL supplementation promotes the differentiation and therapeutic and immunomodulatory potential of hMSCs. Indeed, a three times higher proliferative potential of hPL-supplemented cultures compared to MSCs cultivated with FBS was reported [39,41,50,51]. In this regard, an increase in cell proliferation in MSCs cultivated with hPL compared to ones cultivated with FBS was confirmed also by the higher MSC collection [19,41]. The increased in hMSC growth induced by hPL could probably be attributed to growth factors present in the platelet lysate, thus, leading to the upregulation of the cell cycle and proteins necessary for DNA replication, as well as the downregulation of genes involved both in development and cell differentiation [52].

Despite the positive effect on cell proliferation, the use of hPL can induce the expression of some hMSC surface molecules, including DNAM-1 ligand PVR, Nectin-2, the NKG2D ligand ULBP3, the adhesion molecules CD49d and αvβ3 and fibroblast-associated protein, leading to a reduction in their immunosuppressive capacity. Therefore, these data could suggest some limitations of hPL use in MSC expansion [23].

Although human-blood-derived media showed advantages in MSC expansion, also retaining their multilineage potential, conflicting results regarding MSC differentiation were found. A similar adipogenic differentiation potential was observed in BM-MSCs cultivated with hPL and FBS. In contrast, the integration of BM-MSCs with hPL showed a greater chondrogenic differentiation potential than FBS-treated MSCs [53].

Another study demonstrated that MSCs supplemented with hPL showed a greater osteogenic differentiation and a reduction in adipogenic ones [51].

To replace the use of animal serum in human AD-MSC cultures, the neurotrophic potential of hPL was demonstrated. When used alone, hPL did not induce neurite elongation in human AD-MSCs compared with the FBS cultivated group. In contrast, human AD-MSCs showed a significant increase in neurite growth when cultivated on different ECM substrates (laminin and fibronectin) and hPL. In particular, it was reported that hPL increased the proliferation of AD-MSCs, as well as the neutrophic properties as shown by the release of BDNF. Therefore, hPL could enhance neuron–matrix interactions so that it could be applied in neuronal regeneration [54].

Likewise, a potential strategy for nerve regenerative medicine could be the use of hPL for AD-MSC cultures. In this regard, Palombella et al. demonstrated the neurogenic potential of human AD-MSCs cultivated with hPL. Human AD-MSCs induced with hPL showed a higher proliferation rate and a more elongated morphology compared to AD-MSCs cultivated with FBS, instead characterized by a fibroblast-like morphology. Additionally, to evaluate the effects on neural regeneration, human AD-MSCs induced with hPL were cocultivated with dorsal root ganglia (DRG) from Sprague Dawley (SD) rats. In this regard, an increase in the neurite length and axonal area was shown in human AD-MSCs cocultivated with DRG compared to the FBS group. Although the mechanisms were not fully elucidated, overall, the direct contact between human AD-MSCs cultivated with hPL and DRG led to a significant improvement in neuronal regeneration, thus, avoiding possible zoonotic problems that can characterize culture conditions with FBS [55].

Moreover, it was reported that hPL improved the neurotrophic potential of AD-MSCs. In fact, hPL promoted axonal growth four times more than AD-MSCs integrated with FBS [56].

Despite variations between donors making the standardization of hPL-supplemented culture conditions difficult, overall, the use of hPL may be the most effective human-blood-derived substitute to replace FBS in the expansion of serum-free MSCs [57]. In fact, hPL could also be autologous by the patient to reduce the exposure to xenogeneic/allogenic compounds and any immunological reactions following transplant. In this regard, several studies that used hPL for the growth and differentiation of hMSCs proved it to be safe for potential administration in patients [39,41,57].

The results discussed thus far are presented in Table 3.

### 3.4. Chemically Defined Medium in MSC Cultures

In order to support the in vitro growth of hMSCs in the absence of animal serum, media chemically integrated with growth factors and nutrients were developed. Unlike a serum which contains a set of growth factors and other agents of different molecular weight, a chemically defined medium must contain these factors individually, as well as in precise concentrations to mimic the properties of a serum on hMSC cultures [39]. Therefore, the design of an ideal serum-free culture method by using a chemically defined medium must promote both the adhesion and expansion of MSCs in the first step to exclude contaminating cells from the culture dish and avoid early replicative senescence and cell morphological changes [19].

Generally, a chemically defined medium can be created from a basal culture medium, including DMEM α-MEM selectively integrated with components necessary for the cultivation of the required cell type. Consequently, the concentration and selection of growth factors could influence the expansion and differentiation of MSCs [58].

In this regard, the effects of SFM supplemented with growth factors on the expansion of hMSCs were tested. Several studies demonstrated that SFM combined with growth factors, alone or in combination, promoted hMSC expansion [59,60,61,62].

Moreover, supplementing SFM with recombinant human PDGF-BB, bFGF and TGF-β1 led to an expression profile and a phenotype and differentiation characteristics of BM-MSCs similar to those grown in a standard medium supplemented with FBS [63].

Additionally, it was reported that the increase in bFGF in the medium induced hMSCs towards osteogenic differentiation [58].

To improve cell adhesion as well as the culture of hMSCs, apart from growth factors, the use of attachment factors such as fibronectin or coating the surface with gelatin, an alginate or nanoscaffolds can be integrated into the medium or coated on the culture surface. Additionally, for long-term hMSC culture, hormones such as hydrocortisone in appropriate concentrations can be integrated into the chemically defined medium [64].

Moreover, molecules with a cofactor role such as biotin, ethanolamine and microelements such as selenium and iron integrated in the culture media of hMSCs can induce changes in cell proliferation and differentiation by gene expression regulation [65].

Therefore, to ensure optimal cell growth, a chemically defined medium requires quality controls and media formulations that can greatly influence the morphology, growth and frequency of primary and passaged cultures [66].

Moreover, to prevent excessive cell growth and the production of more homogeneous MSCs, systematically defined formulations are developed, so that the medium can have a more defined profile capable of improving the efficacy of MSCs for possible clinical applications according to protocols and desired properties [67].

To make the culture media more specific for the cells, the use of SFM has led to a number of advantages, including both controlled and defined culture conditions as well as the elimination of potential microbiological contamination [68].

Given their capacity to differentiate into different cell types, including neural lineages, the use of MSCs could be useful for the treatment of neurodegenerative diseases [69,70]. Consequently, it is necessary to develop solutions that allow for replacing the xenogenic substances added to culture media to avoid problems of immunogenic and infectious transmission [71].

Several studies have evaluated the supplementation of a basal medium with growth factors or chemical compounds to stimulate proliferation and neuronal differentiation.

Faghih et al. demonstrated the effects of serum-free conditions on AD-MSC neuronal differentiation. To improve dopaminergic differentiation, AD-MSCs were cultivated with neurobasal medium B27 and serum-free conditions and compared with low-serum condition results obtained previously. Although both differentiation protocols demonstrated similar effects, morphological changes in the neuronal development and a greater number of genes associated with neuronal and dopaminergic differentiation were shown in serum-free conditions compared to low-serum conditions. In detail, it was reported that AD-MSCs cultivated in serum-free conditions reported the upregulation in the expression levels of NSE, GLI1, EN1 and NURR1 markers, which are important in the development and differentiation of dopaminergic neurons. Moreover, in serum-free neurobasal medium conditions, an upregulation in the expression levels of tyrosine hydroxylase (TH) and VMAT2, respectively, involved in catalyzing the synthesis and release of dopamine, was highlighted. Therefore, the study demonstrated that serum-free culture conditions may be useful in regenerative medicine by promoting the differentiation of AD-MSCs toward dopaminergic neurons [72].

The proliferative, angiogenic and neutrophic properties of AD-MSCs and BM-MSCs cultivated in defined xeno- and serum-free conditions were investigated by Brohlin et al. The authors compared them with MSCs cultivated in a medium with fetal calf serum (FCS). The serum-free and xeno-free media culture conditions led to an increase in proliferation as well as an improvement in neutrophic and angiogenic activity in AD-MSCs and BM-MSCs, as shown by the expression levels of BDNF, VEGF-A and angiopoietin-1. Noteworthy is that the serum-free and xeno-free media cultures enhanced the neurite growth in AD-MSCs and promoted a network of capillary-like tubes in BM-MSCs [73].

The growth and differentiation properties of a plasma-derived xeno-free supplement for cell culture (SCC) were evaluated by Díez et al. in human BM-MSCs. Two cell lines of human BM-MSCs were cultivated with different media formulations and compared to BM-MSCs induced with basal medium 2 (BM2) and SCC. The results of the study demonstrated that SCC integration maintained the ability of human BM-MSCs to differentiate into various cell lineages, including neurons, adipocytes, chondrocytes and osteoblasts, as evidenced by the microscopic analysis. Both media supplemented with 15% SCC and BM2 preserved the ability of MSCs to differentiate into neurons, as shown by the presence of elongated axons and dendrites. Therefore, SCC can be considered a stable and safe xeno-free supplement that allows for the growth and differentiation of human BM-MSCs [74].

Multilineage-differentiating stress-enduring (Muse) cells are non-tumor endogenous stem cells that consist of a small percentage of MSCs able to generate all three germ states. To induce differentiation towards a neural phenotype and regenerative properties of human Muse cells, Uchida et al. cultivated Muse cells isolated from human BM-MSCs under serum-free conditions and administered them into the brains of mice with subacute lacunar stroke. The study results showed that 8 weeks after transplant, most Muse cells were differentiated into neuronal cells as shown by the vesicular glutamate transporter (VGLUT) marker, a glutamatergic neuronal marker and synaptophysin. Overall, the study results demonstrated that appropriate culture conditions can direct the fate of MSCs towards a neural phenotype so that they can be potentially applied in neurodegenerative diseases [75].

The ability of DP-MSCs to differentiate into neuron-like cells was investigated by Zainal Ariffin et al. To evaluate the morphological changes, MSCs were cultivated in SFM without supplementation with growth factors. The study results showed a neural-like phenotype of DP-MSCs evaluated by MAP2 expression and neuronal marker activation nestin and Tub3. Therefore, the study provided evidence for the direct differentiation of DP-MSCs in serum-free conditions and without growth factors [76].

The ability of MSCs from human exfoliated deciduous teeth (SHEDs) to differentiate into neurons and oligodendrocytes under serum-free culture conditions was also shown by Matsubara et al. The authors demonstrated that CM from SHEDs (SHEDs-CM) obtained under serum-free conditions included factors capable of promoting functional recovery following neural damage. In detail, it was shown that SHEDs-CM led to an anti-inflammatory response inducing the M2 state of macrophages. Additionally, the secretome analysis of SHEDs-CM reported that anti-inflammatory action was induced by monocyte chemoattractant protein-1 (MCP-1) and the secreted ectodomain of sialic acid-binding Ig-like lectin-9 (ED-Siglec-9), which by binding to CC chemokine receptor 2 (CCR2) of MCP-1 modifies its signaling to induce the anti-inflammatory action and reduce the tissue damage. Indeed, MCP-1/ED-Siglec-9 preserved axons and reduced the neural damage, thus, promoting functional recovery in vivo [77].

The results discussed thus far are presented in Table 4.

### 3.5. In Vitro Studies That Demonstrated the Effects of Growth Factors in Neuronal MSC Cultures

The effects of the use of specific xeno-free media on MSC differentiation were evaluated by Elgamal et al. in human AD-MSCs cultivated in SFM. To induce neuronal or glial differentiation, blood-derived formulations rich in platelets, including human-activated pure platelet-rich plasma (P-PRP) and advanced platelet-rich fibrin (A-PRF) in SFM supplemented with human cerebrospinal fluid (CSF), were added to MSC cultures and compared with the control group cultured with FBS 10%. The study results indicated that P-PRP and A-PRF induced AD-MSC neurogenic proliferation and differentiation. Moreover, it was shown that P-PRP and A-PRF, both at 20%, were more potent inducers than 10% of FBS. Similarly, 10% or 20% activated P-PRP was a more potent inducer than 10% or 20% A-PRF, respectively. It was also found that media with 10% CSF and 10% PRP promoted glial differentiation, while only 10% PRP induced a neuron-like phenotype. Therefore, to increase the proliferative rate of AD-MSC, both activated P-PRP and A-PRF can replace FBS. Although there are several protocols for differentiating MSCs, the use of multiple growth factors may have a better effect. In this regard, PRP contains numerous growth factors, including BDNF, NGF, PDGF, vascular endothelial growth factor (VEGF), TGF-β and insulin-like growth factor-1 (IGF-1). Moreover, CSF also contains growth factors such as GDNF, BDNF and bFGF. Noteworthy is that the combination of PRP and CSF inhibited neuronal differentiation and stimulated the glial differentiation as shown by the downregulation of nestin and microtubule-associated protein 2 (MAP2) levels and increase in GFAP expression levels. AD-MSCs cultured with PRP 10% showed a neurogenic differentiation, evaluated by the increase in MAP2 and nestin, as well as by the downregulation of GFAP levels [78].

AD-MSC neural differentiation was also investigated by Mannino et al. using a conditioned medium (CM) from the retinal pigment epithelia (RPE) cell line ARPE-19. To investigate neuronal differentiation, the authors used SFM and physiological conditions similar to those of the eye. Several culture conditions, including AD-MSCs cultivated in DMEM/FBS, AD-MSCs grown in serum-free DMEM, AD-MSCs cultivated in serum-free DMEM/F12 and AD-MSCs grown in a CM from ARPE-19, were used. AD-MSCs cultured in serum-free conditions demonstrated a lower viability and proliferation. Noteworthy is that AD-MSCs with ARPE-19 CM showed a significant increase in neuron-specific enolase (NSE) and GFAP. Instead, a less significant increase in nestin and protein gene product 9.5 (PGP9.5) was highlighted. Further studies are needed to clarify the mechanisms by which AD-MSCs differentiate towards a neural phenotype. In this regard, the presence of extracellular vesicles in ARPE-19 CM cannot be excluded [79].

A method to induce AD-MSC differentiation into neural progenitor cells was performed by Peng et al. The authors cultured human AD-MSCs in a defined induction medium under serum-free conditions, supplemented with EGF, bFGF, N2 and B27. After 12 h of induction, neural progenitor cells that grew as neurospheres could either self-renew and form secondary neurospheres, or be induced to a neuronal phenotype and glial cells. Additionally, the secretome analysis of neurospheres demonstrated that AD-MSCs could be a source of neural progenitor cells, as reported by the increase in the expression levels of neurogenic and angiogenic cytokines, including BDNF, bFGF, GDNF and VEGF. Therefore, AD-MSC-derived neurospheres could be an important source of neural progenitor cells with a high regenerative potential [80].

The differentiation potential of BM-MSCs towards a neural phenotype was investigated by Arnhold et al. Human BM-MSCs were cultivated in SFM supplemented with B27 and growth factors, including EGF, bFGF and heparin, to form three-dimensional cell aggregates. Moreover, to verify the neural differentiation, human BM-MSCs were incubated in a serum-free differentiation medium integrated with dimethyl sulfoxide (DMSO) and forskolin. Overall, the BM-MSCs grown under serum-free conditions demonstrated neural characteristics, confirmed by the increase in nestin and glial precursors A2B5 compared to standard conditions [81].

The neurogenic and gliogenic capacity of dental pulp-derived MSCs (DP-MSCs) using a serum-free protocol was demonstrated by Luzuriaga et al. To demonstrate neurogenic potential, the authors cultured human DP-MSCs for 2 weeks under different culture conditions: medium plus FBS, SFM and SFM supplemented with BDNF and neurotrophin 3 (NT-3) for 7 days. The results showed that DP-MSCs cultured in serum-free conditions overexpressed the neurotrophin receptor genes NTRK2 (TrkB) and NTRK3 (TrkC) compared to the FBS group. Moreover, the presence of BDNF and NT-3 in the serum-free group improved the neural crest (NC) progenitor properties of DP-MSCs, as reported by the increase in the p75 neurotrophin receptor (p75^NTR^), human natural killer-1 (HNK-1) and also by the overexpression of key markers in the maintenance of pluripotency, including NANOG, OCT4 and SOX2. Moreover, BDNF and NT-3 combined in SFM led the DP-MSCs to differentiate towards a neuronal and glial lineage, as shown by the positivity for DCX, NeuN, S100ß and p75^NTR^ markers. Therefore, the study suggested that BDNF and NT-3 are capable of reprogramming DP-MSCs cultivated in SFM into neurogenic and gliogenic NC progenitors [82].

Additionally, Kawase-Koga investigated the DP-MSC ability to proliferate and differentiate into neurons in xenon-/serum-free conditions. To induce the neurosphere formation, human DP-MSCs were isolated and cultivated for 14 days in xeno-/serum-free conditions. Additionally, to induce neuronal differentiation, neurospheres were grown in a differentiation medium supplemented with bFGF and EGF. In this regard, 21 days after neuronal differentiation, it was found that neural cell markers nestin, β-3 tubulin, SOX2, Vimentin and neurofilament-M were more expressed in neurospheres derived from DP-MSCs grown in xeno-/serum-free conditions compared to the group cultured with FBS [83].

Furthermore, Bonnamain et al. demonstrated the effects of serum-free culture conditions on neural DP-MSC differentiation. To evaluate the ability to proliferate and differentiate towards a neural phenotype, human DP-MSCs were separated into adherent (ADH) and non-ADH cells and cultivated for 12 h in a basal medium supplemented with an N2, bFGF, EGF and heparin solution. The study results showed that both ADH and non-ADH MSCs contained neuronal or oligodendrocytic progenitors. In detail, ADH cells appeared similar to fibroblasts or nodules with a spherical morphology and extensive processes. In this regard, ADH DPMSCs expressed neural markers, including nestin, β-3 tubulin and neurofilament-M, and the oligodendrocyte marker proteolipid-protein-1 (PLP1). In the same way, non-ADH DP-MSCs that grew as spheroids showed transcripts of nestin, β-3 tubulin, neurofilament-M and PLP-1. Despite the levels of nestin, β-3 tubulin and NF-M were similar, with a greater differentiation towards oligodendrocytes of ADH MSCs compared to non-ADH cells being reported, as confirmed by the higher transcription of PLP-1 levels. Additionally, it was also shown that non-ADH DPMSCs could form spheroid structures more oriented towards an odontoblastic phenotype compared to ADH DPMSCs [84].

The effects on DP-MSC differentiation towards different lineages and, in particular, the neuronal one, were investigated by Karbanova et al. To induce neuronal proliferation and differentiation, human DP-MSCs as a monolayer or spheroids were grown for 3 weeks in serum-free conditions and a neurogenic medium supplemented with B27, bFGF, EGF and bone morphogenetic protein (BMP)-2, involved in the differentiation of neurons and oligodendrocytes from neural stem/stromal cells. The results of the study showed that DP-MSCs cultivated as a monolayer led to Upregulation of nestin, A2B5 and radial glial cells (RC2). Furthermore, DP-MSCs grown in serum-free conditions and a neurogenic medium supplemented with BMP-2 lost the ability to proliferate, and also caused a significant increase in β3-tubulin and PanNF levels. Contrarily, the neuronal differentiation process of DP-MSCs was inhibited when serum 1% was added [85].

Fatima et al., evaluated the phenotypic and molecular changes as well as the differentiation of human DP-MSCs in different lineages and, in particular, the neuronal one in serum-free conditions. To induce neurogenic stimulation and analyze neurospheres, DP-MSCs were cultivated for 21 days in SFM integrated with EGF and fibroblast growth factor (FGF). To determine the DP-MSC neurogenic potential, ADH and non-ADH neurospheres were induced with retinoic acid (RA), as well as the mitogenic factors EGF and FGF being removed. Noteworthy is that non-ADH neurospheres demonstrated a greater potential to generate neurons as reported by well-developed axons and dendrites compared to ADH neurospheres. Additionally, non-ADH neurospheres showed a higher expression level of β-3 tubulin. Therefore, the study results encourage the development of regenerative strategies that involve DP-MSCs cultivated under serum-free conditions [86].

Several protocols of cholinergic neuron differentiation were evaluated by Kang et al. using human DP-MSCs. In the first protocol, DP-MSCs were cultivated in SFM containing β-mercaptoethanol (β-ME) for 24 h and incubated with NGF for 6 days. In the second protocol, DP-MSCs were grown in SFM supplemented with tricyclodecan-9-yl-xanthogenate (D609) for 4 days, while, in the third protocol, DP-MSCs were cultivated in SFM containing bFGF, forskolin, sonic hedgehog (SHH) and RA for 7 days. All protocols led to DP-MSC differentiation towards a neuronal-like phenotype as shown by the upregulation of cholinergic neuronal markers, including choline acetyltransferase (ChAT), MAP2, HB9, ISL1 and BETA-3. In particular, it was found that cholinergic neurons differentiated according to protocols II and III showed a marked increase in cholinergic neuronal markers. Overall, it was shown that the combined use of growth factors, SHH, RA and serum-free conditions in MSC neuronal differentiation protocols led to an increase in neuronal markers, as well as the secretion of neurotransmitters [87].

The neuronal differentiation of human umbilical cord blood-derived MSCs (hUCB-MSCs) cultured in SFM conditions, as well as the underlying mechanisms that involved NOTCH signaling were also investigated by Venkatesh et al. To induce neuronal differentiation, hUCB-MSCs were cultivated in SFM supplemented with EGF and FGF. The subsequent immunofluorescence analysis showed an increase in nestin and SOX2 in neurospheres generated by hUCB-MSCs. Moreover, to investigate the NOTCH signaling involvement in the differentiation of MSCs towards a neuronal phenotype, the authors used the NOTCH inhibitor N-[N-(3,5-difluorophenacetyl)-l-alanyl]-S-phenylglycinet-butyl ester (DAPT). The results of the study demonstrated that DAPT significantly reduced the number of neurospheres formed, in association with a downregulation of nestin and SOX2. Moreover, DAPT led to the inhibition of Mushashi-1, thus, confirming the role of NOTCH signaling in neuronal proliferation and differentiation. Additionally, it was also shown that DAPT downregulated Notch target genes, including HES1 and HES5, the neuronal markers MAP2 and NEFH as well as glial markers GFAP, GLUL and MBP. Therefore, the study reported that appropriate culture conditions together with NOTCH signaling can be useful to improve the maturation, self-renewal and neuronal differentiation of MSCs [88].

The involvement of Notch signaling in the neurogenic differentiation of human periodontal ligament-derived MSCs (hPDL-MSCs) under serum-free conditions, was shown by Osathanon et al. To induce neurogenic differentiation, hPDL-MSCs were cultivated in a neurobasal medium supplemented with EGF and bFGF. Subsequently, the neurospheres were induced with RA for 7 days and neurogenic differentiation was confirmed by β3-tubulin, a neurofilament as well as by the expression levels of Sox2 and Sox9. In order to evaluate the role of Notch signaling in neuronal differentiation, hPDL-MSCs were cultured on a Jagged-1-modified surface, because Jagged-1 is the Notch ligand. These hPDL-MSCs showed an increased expression of the Notch signaling target genes *Hes-1* and *Hey-1.* Moreover, hPDL-MSCs on surface-bound Jagged-1 under serum-free conditions showed multiple neurite-like extensions and an increased gene expression of neurogenic markers. Additionally, the role of Notch signaling was also shown by pharmacological or genetic approaches that attenuated the formation of neurospheres. Indeed, the use of DAPT, a potent gamma-secretase inhibitor, prevented the nuclear Notch cleavage and reduced the volume of neurospheres compared to hPDL-MSCs not treated with DAPT. Moreover, the neurospheres treated with DAPT reported a reduction in Hes-1, Hey-1 as well as Sox2 and β3-tubulin levels, thus, confirming the role of Notch signaling in hPDL-MSCs neuronal differentiation [89].

The effects on the neuronal differentiation of human olfactory ecto-MSCs (hOE-MSCs) under serum-free conditions were also evaluated by Hamidabadi et al. To induce proliferation as well as differentiation, hOE-MSCs were cultivated for 2 days in SFM supplemented with B27, bFGF and EGF. Additionally, cells were incubated for 6 days in neurobasal medium integrated with bFGF, FGF8b, ascorbic acid and SHH. The results of the study showed that cells differentiated from hOE-MSCs expressed motor neuron-like markers, including Islet-1, ChAT and HB9 [90].

The results discussed thus far are presented in Table 5.

### 3.6. In Vitro Studies That Reported the Effects of Chemical Agents in MSC Cultures

Tao et al., demonstrated the potential of the neuronal differentiation of human BM-MSCs under a serum/feeder cell-free condition by the combined use of bFGF, EGF and PDGF. This was compared with chemical inducing agents, including DMSO and butylated hydroxyanisole (BHA). Human BM-MSCs exposed to bFGF, EGF and PDGF and cultivated under a serum/feeder cell-free condition showed a neural morphology as confirmed by the presence of neuronal markers, including neurofilament-M, β-3 tubulin and NSE. Moreover, it was reported that although BM-MSCs differentiated towards neuronal lineage, the treatment with DMSO and BHA did not induce prolonged neuronal MSC differentiation compared to the group treated with growth factors. Therefore, the study results suggested that the serum/feeder cell-free condition could be an appropriate culture condition to induce BM-MSC neuronal differentiation [91].

The differentiation potential of hMSCs towards auditory neurons and hair cells was shown by Durán Alonso et al. To induce neuronal differentiation, primary cultures of human BM-MSCs were induced into an intermediate neural progenitor stage following two different culture methods. BM-MSCs were cultivated as a monolayer for 24 h in high-glucose DMEM plus FBS 10% and supplemented with β-ME. Subsequently, still in the same medium, the cells were cultivated for 72 h and supplemented with RA. Furthermore, the cells were induced in SFM for 12–15 days. During the first 5 days, NT-3 and bFGF were added to the medium, then bFGF was replaced with BDNF, while the spheres derived from hMSCs were obtained using two protocols. To form the spheres, hMSCs were cultivated for 3 days in SFM supplemented with EGF and bFGF, while in the second protocol, hMSCs were grown in high-glucose DMEM with FBS 10% and supplemented with RA for 5 days. When neural progenitors derived from non-ADH hMSCs cultured in SFM were exposed to EGF and RA, this resulted in hair-cell-like cells, while hMSCs resulted in auditory neuron-like cells when treated with growth factors, including NT-3, BDNF, RA and SHH [92].

The differentiation towards a dopaminergic-like neuronal phenotype was investigated by Barzilay et al. in human BM-MSCs. To induce MSC neuronal differentiation, BM-MSCs were cultivated in SFM containing grown factors. Additionally, cyclic adenosine monophosphate (cAMP), 3-isobutyl-1-methylxanthine (IBMX) and ascorbic acid were added. Moreover, the medium was enriched with BDNF, GDNF, neurturin and NT-3. Noteworthy is that high levels of cAMP were involved in neuronal differentiation, while the presence of ascorbic acid was directly related to dopaminergic differentiation. Overall, the study demonstrated that BM-MSCs were differentiated towards a dopaminergic neuronal phenotype, as reported by a significant increase in the expression levels of TH and Nurr 1, as well as by the reduction in the gamma-aminobutyric acid (GABA)ergic marker expression [93].

The potential of extracellular matrix proteins, including proteins-fibronectin, collagen-1, collagen-IV, laminin-1 and laminin-10/11, to induce the neuronal differentiation of human BM-MSCs was investigated by Mruthyunjaya et al. The study demonstrated that only laminin-1 led to the development of neurites and cell bodies in BM-MSCs cultivated in serum-free conditions. In detail, it was reported that laminin-1 induced the neurite growth in BM-MSCs through the activation of FAK-MEK/ERK signaling pathways involved in survival and cell differentiation. Moreover, it was found that the FAK-MEK/ERK signaling pathway activation was mediated by integrin alpha6beta1. Therefore, the study results demonstrated that under serum-free conditions, laminin-1 could induce neurite growth in MSCs through the FAK-MEK/ERK signaling pathway activation [94].

Additionally, Suon et al., demonstrated the effects on the neuronal differentiation of chemical agents that promote the increase in cAMP levels in BM-MSCs. The results of the study reported that human BM-MSCs incubated with mediums containing 4β-12-O-tetradecanoylphorbol 13-acetate (TBA), forskolin and the phosphodiesterase inhibitor IBMX led to the differentiation of BM-MSCs towards a neuronal-like phenotype. In this regard, it was observed that BM-MSCs changed morphology from being flattened towards that of bipolar cells with branched processes. Additionally, the study of the time course of differentiation showed a rapid (1–4 h) and transient (24–48 h) change of human BM-MSCs, to spontaneously return to the undifferentiated state. The neural differentiation of MSCs was also confirmed by the increased expression levels of NSE, GFAP, β3-tubulin and neurofilament. On the contrary, a reduction in fibronectin (highly expressed in the undifferentiated state) was observed after the differentiation of BM-MSCs. Despite the changes of mRNA and protein levels induced by the addition of actinomycin D and cycloheximide, the neural-like morphology of BM-MSCs was not altered, thus, confirming that mechanisms of morphology acquisition and biochemical composition were independent [95].

The effects on MSC neural differentiation were also investigated by Ayala-Grosso et al. The authors observed the phenotype and expression of proteins involved in the neural differentiation of human olfactory mucosa–derived MSCs (hO-MSCs) under serum-free conditions and growth factors. Under serum-free culture conditions, hO-MSCs expressed olfactory basal progenitor antigens and mostly neural progenitor cells, as confirmed by transcription factors Oct 3/4, Sox-2, Mash-1 and neurotrophins BDNF, NT3 and NT4, respectively. To induce neural differentiation, hO-MSCs were seeded in SFM supplemented with N2, RA and forskolin. In this regard, it was shown that SFM led to the differentiation of hO-MSCs into neurons and astroglial cells [96].

The MSC neuronal differentiation was also studied by Jiang et al. using human first-trimester MSCs (fMSCs) cultivated on polysaccharide nanofiber scaffolds in SFM supplemented with SHH and RA. After 7 days of fMSC culture, the cells differentiated towards a motor neuron lineage and also exhibited an elongated morphology. Moreover, a significant upregulation of motor neuron markers, including oligodendrocyte lineage transcription factor 2 (Olig 2), HB9 and ChAT, was found. Overall, the study demonstrated that scaffolds composed of polysaccharide nanofibers under serum-free conditions induced MSCs towards a motor neuron-like phenotype [97].

The effects on MSC differentiation towards a neural phenotype were demonstrated by Lei et al. To perform the study, BM-MSCs were induced with different cultivation protocols, including SFM supplemented with N2 and B27, SFM and β-ME, and cocultivation with murine astrocytes. The results of the study showed that all the treatments led to the differentiation of BM-MSCS towards a neural phenotype, as reported by the morphological changes and by the levels of neuronal markers. Moreover, it was reported that astrocyte culture resulted in the BM-MSCs differentiation mainly towards glial cells, as demonstrated by the presence of GFAP. Therefore, the presence of astrocytes, probably thanks to the secretion of some factors, could direct the BM-MSCs to differentiate towards glial cells rather than neurons [98].

Additionally, Khanabdali et al., demonstrated the effects of chemical agents on the cardiomyogenic and neuronal differentiation of rat BM-MSCs. BM-MSCs were treated with zebularine and 5-azacytidine for 24 h, after which MSCs formed myotube-like structures after 10 days in culture. Moreover, higher levels of troponin-T and cardiogenic transcription factors, such as GATA-4 and NK2 transcription factor-related locus 5 (Nkx2.5), were found, while to induce neurogenic differentiation, BM-MSCs were first cultivated with serum-enriched DMEM and β-ME for 24 h and replaced with serum-free DMEM supplemented with β-ME for 3 h. Noteworthy is that the second induction with β-ME and serum-free conditions led to BM-MSC differentiation towards neuron-like cells, as shown by the presence of axon-like and dendrite-like processes. Moreover, the BM-MSC differentiation towards a neuronal phenotype was demonstrated by the presence of neuronal markers, including nestin, MAP2, Tau and Nefl. Therefore, the study reported that BM-MSCs were differentiated towards a neuronal phenotype under serum-free conditions and without the use of growth factors or cytokines [99].

The effects on BM-MSC neural differentiation were also investigated by Rooney et al. To perform the study, rat BM-MSCs were cultivated under serum-free conditions and supplemented with forskolin, used to promote the intracellular increase in cAMP. The study results demonstrated that forskolin induced BM-MSCs towards a neural-like morphology, as shown by the presence of neurite-like processes and NSE and neurofilament 200 markers, as well as by the significant increase in β-3 tubulin. Noteworthy is that, after 24 h of forskolin-induction, the BM-MSCs showed the original morphology, confirming a half-life of approximately 6–8 h and a transient effect of forskolin on neural differentiation. Moreover, the effects on the morphological changes of MSCs under serum-free conditions were confirmed by 8 bromo-cAMP use. Indeed, 8 bromo-cAMP induced in BM-MSCs both a neuronal-like morphology and an increase in β-3 tubulin levels. Additionally, to evaluate cell survival and viability, the authors cultivated BM-MSCs under serum-free conditions with the supplementation of ascorbic acid 2 phosphate. The treatment with ascorbic acid 2 phosphate improved cell survival and reduced the apoptosis levels in BM-MSCs compared to the FBS-treated group. Overall, the study reported a transient effect of compounds that promoted the increase in cAMP on the cell morphology, rather than a true differentiation towards a neural phenotype of MSCs [100].

To test the effects on neuronal differentiation in murine AD-MSCs and embryonic stem cells (ES), Taha et al. used a medium containing a synthetic serum known as a knockout serum replacement (KOSR) and compared its potential with low-serum conditions. Moreover, the authors evaluated the neurogenic potential of β-ME, which was used as an inducer for the AD-MSC neuronal differentiation. To induce neuronal differentiation, AD-MSCs were cultivated according to two different culture conditions: DMEM plus 15% KOSR and DMEM plus 4% FBS, while the effects of 0.1 or 1 mM β-ME on the AD-MSCs neuronal differentiation were compared with the control group cultured without β-ME. The study results demonstrated that KOSR induced the MSC neural differentiation, as evidenced by the presence of MAP2. Furthermore, both in low-serum and KOSR conditions, AD-MSCs were differentiated into neuron-like cells, as shown by neuronal markers Pax6 and nestin. Moreover, it was shown that β-ME 0.1 mM supplemented with FBS 4% improved the neuronal differentiation of AD-MSCs. Conversely, adding β-ME in KOSR conditions led to a reduction in neuronal differentiation. In particular, it was reported that 1 mM β-ME induced neurotoxicity in AD-MSCs, especially when used with KOSR-containing media. Therefore, the study results demonstrated that the KOSR-supplemented medium could be useful for the direct differentiation of MSCs towards a neuronal phenotype [101].

The results discussed thus far are presented in Table 6.

## 4. Extracellular Matrix

To cultivated MSCs on substrates capable of reproducing a neural microenvironment and providing signal capable of starting and supporting the cellular functions in the best possible way, as well as overcoming the limits regarding the use of monolayer and 2D cultures, biomaterials were studied. Since the neural microenvironment is insufficiently simulated in vitro, the use of biomaterials can potentially reproduce biochemical and biophysical signals derived from the ECM. Therefore, material scaffolds can be developed to mimic cell features and support the intrinsic ability of MSCs to reproduce interactions that occur in brain development [102].

The neural ECM is mainly composed of hyaluronic acid, chondroitin sulfate proteoglycans and heparan sulfate proteoglycans, connecting proteins, heparan sulfate proteoglycans, tenascins, laminins and reelin [103]. Additionally, a low percentage of fibrous proteins, including collagen and fibronectin, shows the aspect of an amorphous reticulum [104].

Several studies demonstrated the effects of neural ECM on cell proliferation and differentiation. Indeed, it was reported that elevated levels of hyaluronic acid promoted the proliferation of astrocytes and a decrease in neural progenitor cells in the injured spinal cord of rats [105,106]. Furthermore, it was observed that the reduction in chondroitin sulfate proteoglycans led to a reduction in neural progenitor cells, orienting them towards glial differentiation [107]. In contrast, it was reported that the addition of exogenous chondroitin sulfate proteoglycans promoted the proliferation of neural progenitor cells [108]. Overall, the data suggested that levels of chondroitin sulfate proteoglycans modulate neuronal/glial differentiation and the proliferation of neural progenitor cells [107].

Consequently, it is necessary to improve the cultivation of MSCs in 3D and the interaction between cells, as well as the mechanisms underlying it, to combine scaffolds and cells as a potential application for tissue engineering in the brain. In this regard, encouraging results have been and continue to be obtained. Indeed, a reduction in cellular aging was shown in hBM-MSCs grown on a denatured collagen matrix, as well as an improvement in cell proliferation and an increase in the maintenance of the osteogenic differentiation potential for a longer amount of time [109]. In the same way, it was reported that the maintenance of adipogenic and osteogenic differentiation potential in hBMSCs cultivated on a denatured collagen type I matrix, thus, demonstrating the important role of ECM in hMSC cultures [110,111].

Therefore, apart from collagen, the use of synthetic hydrogel networks consisting of polyethylene glycol (PEG) or polyethylene glycol fumarate combined with molecules that allow the adhesion of hMSCs are being shown to be effective in stem cell cultures [112,113]. Consequently, if coupled to serum-free or xenon-free culture conditions, these approaches used for the expansion of MSCs could potentially be useful for administration to patients.

## 5. Future Possible Solutions

Despite the success obtained in experimental studies that used alternatives to animal serum in MSC cultures, further studies are needed for MSC therapeutic applications. Regardless of the present xenon-free media consisting of defined components, there are limitations for their use in MSC cultivation [114,115].

Blood derivatives, such as human serum, hUCBS and hPL, consist of undefined components. Moreover, the batch-to-batch variability as well as the possible presence of pathogens not detected by screening are potential limits for their use in future clinical applications [114].

Among the alternatives to animal serum, when used in combination with MSCs cultivated on different ECM substrates, hPL promoted a high neurogenic and proliferative potential in MSCs [55,56]. Although the standardization of culture conditions supplemented with hPL is currently difficult, it was shown that autologous hPL may be safe for expansion and the future administration of MSCs in patients [57]. Moreover, since human blood derivatives can influence the immunophenotypic, genotypic and functional characteristics of hMSCs, to determined standardized protocols of MSC cultivation, it might be necessary to use a proteomic analysis to identify the factors as well as the signaling mechanisms that influence the hMSC cultures. Additionally, the large-scale production of these factors present in the blood derivatives could be the next step to induce the expansion and differentiation of MSCs, so as to development a standard and defined medium potentially useful for therapeutic applications [19].

Nowadays, there are no chemically defined serum-free mediums; thus, researchers are investing in human supplements and biomaterials to obtain safe and standardized MSC cultures as well as alternatives to using animal serum.

As shown in preclinical studies, the use of growth factors alone or combined could be a viable way to substitute animal serum for growth and the neuronal differentiation of MSCs [78,80,82,83]. Although it is necessary to standardize these methods and also to choose the cell line to be cultivated, on the contrary, the use of chemical agents integrated in SFM could lead to a transitory effect in the differentiation of MSCs towards a neuronal-like phenotype [95]. Additionally, the possible neurotoxicity induced by some chemical agents could not only induce differentiation, but also damage the cell. Therefore, methods that preserve both the cell from damage and promote neurodifferentiation are needed.

To test the efficacy of hMSC administration, several clinical trials are currently underway [116,117,118]. In this regard, it was reported that hMSCs expanded in FCS could already be used to generate an immune response in some patients from the first administration with a possible increase in immune response in following hMSC administrations. In fact, in a clinical study conducted for the treatment of osteogenesis imperfecta, cell engraftment was not shown in one patient, as evidenced also by antibodies directed against FCS proteins [117].

Hence, to maintain the proliferative capacity and differentiation potential of hMSCs, protocols able to remove the proteins contained in animal serum are needed. In this regard, researchers are investigating optimal conditions capable of removing up to 99.99% of xenogenic proteins. Indeed, it was shown that MSCs cultivated in 20% FCS induced a humoral response in rats after repeated administration. In contrast, by reducing the proteins contained in the FCS by approximately 100,000 times, it was shown that MSCs maintained the proliferative and multilinear differentiation capacity. Noteworthy is that it was reported that a significant reduction from 7 to 30 mg of proteins contained in FCS was associated with a standard preparation of 100 million, compared to less than 100 ng FCS proteins per 100 million hMSCs [27].

Although further studies are required, the elimination of proteins from animal serum could also be a viable direction for MSC cultures.

## 6. Conclusions

The improvement of culture conditions of MSCs is necessary for their clinical application. One of the obstacles associated with current standard culture methods is the use of animal serum. Several studies have demonstrated the efficacy of using serum-free culture media in the neuronal proliferation and differentiation of MSCs. Furthermore, it was shown that supplementation with growth factors, including bFGF, PDGF and TGF-β1 in SFM, improved the proliferation and neuronal differentiation of MSCs.

To date, further studies are needed to standardize the serum-free culture conditions of MSCs.

## Figures and Tables

**Table 1:** Overview of the in vitro studies that summarize the effects of human serum in MSC cultures.

MSCs	Culture/Differentiation Conditions	Results	References
Human BM-MSCs	HAS 10% in the absence of growth factors and cytokines and FBS 10%	increase proliferation in HAS decrease in the differentiation capacity in HAS group compared to FBS-cultivated MSCs	[24]
Human BM-MSCs	Allogeneic serum 10% and FCS 10%	increase in the proliferation and longer time of hMSC adhesion	[27]

MSCs: mesenchymal stromal cells; BM-MSCs: bone-marrow-derived MSCs; HAS: human autologous serum; FBS: fetal bovine serum; FCS: fetal calf serum.

**Table 2:** Summary of the in vitro studies that reported the effects of human umbilical cord blood serum in MSCs cultures.

MSCs	Culture/Differentiation Conditions	Results	References
Human BM-MSCs	hUCBS 10% and FCS 10%	increase in growth, proliferation and differentiation in BM-MSCs cultivated with hUCBS	[28]
Human BM-MSCs	hUCBS 10% and FBS 10%	Shorter doubling times and a shift in the differentiation potential in BM-MSCs cultivated with hUCBS	[29]

MSCs: mesenchymal stromal cells; BM-MSCs: bone-marrow-derived MSCs; hUCBS: human umbilical cord blood serum; FCS: fetal calf serum; FBS: fetal bovine serum.

**Table 3:** Overview of the in vitro studies that summarize the effects of human platelet derivatives in MSC cultures.

MSCs	Culture/Differentiation Conditions	Results	References
Human AD-MSCs	DMEM/F12 supplemented with 5% hPL and ECM; DMEM/F12 integrated with 10% FBS	increase in cell proliferation and neurite growth in AD-MSCs cultivated with hPL and ECM	[54]
Human AD-MSCs	AD-MSCs cultivated with hPL and cocultivated with DRG from SD rats	increase in neurite length and axonal area was shown in human AD-MSCs hPL induced and cocultivated with DRG	[55]
Human AD-MSCs	5% hPL and 10% FBS	increase of axonal growth in AD-MSCs supplemented with hPL	[56]

MSCs: mesenchymal stromal cells; AD-MSCs: adipose-derived MSCs; hPL: human platelet lysate; ECM: extracellular matrix; FBS: fetal bovine serum; DRG: dorsal root ganglia; SD: Sprague Dawley.

**Table 4:** Summary of the in vitro studies that showed the effects of chemically defined medium in MSC cultures.

MSCs	Culture/Differentiation Conditions	Results	References
Human AD-MSCs	Serum-free neurobasal B27 medium	increase in the development and differentiation of dopaminergic neurons increase of dopaminergic markers, including NSE, GLI1, EN1, NURR1, TH and VMAT2	[72]
Human AD-MSCs and human BM-MSCs	Defined serum-free and xeno-free media	increase in cell proliferation, neutrophic and angiogenic activity increase of BDNF, VEGF-A and angiopoietin-1	[73]
Human BM-MSCs	BM-MSCs cultivated with BM2 and differentiated with a plasma-derived xeno-free supplement for SCC	Both SCC supplemented medium and BM2 promoted the neurodifferentiation of MSCs as shown by the elongated axons and dendrites	[74]
Muse isolated from human BM-MSCs	Serum-free conditions	increase of VGLUT and synaptophysin	[75]
DP-MSCs from 6- to 8-week-old mice	SFM	increase in neural-like phenotype glial differentiation in astrocyte culture increase of MAP2, nestin and Tub3	[76]
SHEDs	Serum-free conditions	increase in anti-inflammatory response through M2 state of macrophages induced by SHEDs-CM	[77]

MSCs: mesenchymal stromal cells; AD-MSCs: adipose-derived MSCs; NSE: neuron-specific enolase; TH: tyrosine hydroxylase; BM-MSCs: bone-marrow-derived MSCs; BDNF: brain-derived neurotrophic factor; VEGF-A: vascular endothelial growth factor-A; BM2: basal medium 2; SCC: supplement for cell culture; VGLUT: vesicular glutamate transporter; DP-MSCs: dental pulp-derived MSCs; SFM: serum-free media; MAP2: microtubule-associated protein 2; SHEDs: human exfoliated deciduous teeth; SHEDs-CM: CM from SHEDs.

**Table 5:** Summary of the in vitro studies that reported the effects on MSCs cultivated in serum-free conditions with the supplementation of grown factors used alone or combined with chemical agents or CSF.

MSCs	Culture/Differentiation Conditions	Results	References
Human AD-MSCs	SFM supplemented with P-PRP and A-PRF at different concentrations of 20, 10, 5, 2.5, 1.25 and 0.6% used alone or in combination with human CSF	Proliferation and neurogenesis induced by P-PRP and A-PRF increase glial differentiation induced by media integrated with CSF and PRP	[78]
Human AD-MSCs	CM from ARPE-19 cultivated in	increase of NSE and GFAP	[79]
Human AD-MSCs	serum-free SFM and defined induction medium, including EGF 20 ng/mL, bFGF 20 ng/mL supplemented with N2 and B27	increase in neuronal differentiation increase of nestin and Sox2	[80]
Human BM-MSCs	SFM supplemented with B27 and with EGF 20 ng/mL, bFGF 20 ng mL and heparin 5 μg/mL MSCs incubated in a serum-free differentiation medium, supplemented with DMSO and forskolin 1 μM	increase in neural morphology increase nestin and A2B5	[81]
Human DP-MSCs	medium plus FBS, SFM and SFM supplemented with BDNF 500 ng/mL and NT-3 20 ng/mL for 7 days	SFM supplemented with BDNF and NT-3 combined led MSCs to differentiate towards a neuronal and glial lineage, as shown by the positivity of markers DCX, NeuN, S100ß and p75NTR	[82]
Human DP-MSCs	Xeno-/serum-free conditions and differentiation in medium supplemented with bFGF 10 ng/mL and EGF 20 ng/mL	increase of β-3 tubulin, SOX2, Vimentin and neurofilament-M	[83]
Human DP-MSCs	ADH and non-ADH cells differentiated for 12 h in a basal medium supplemented with N2, bFGF 25 ng/mL, EGF 10 ng/mL and heparin solution	increase in differentiation towards oligodendrocytes of ADH MSCs, as shown by PLP-1 levels	[84]
Human DP-MSCs	Serum-free neurogenic medium supplemented with B27, bFGF 10 ng/mL, EGF 20 ng/mL and BMP-2 50 ng/mL	increase cells proliferation, β3-tubulin and PanNF levels in serum-free conditions increase nestin, A2B5 and RC2 in DP-MSCs cultivated as monolayer	[85]
Human DP-MSCs	SFM supplemented with EGF 20 μg/mL and FGF 10 μg/mL for 21 days; additionally, neurospheres were induced with RA 0.05 μM and with both EGF and FGF removed	increase axon and dendrite development and β-3 tubulin in non-ADH cells compared to ADH cells	[86]
Human DP-MSCs	SFM containing β-ME 1 mM for 24 h and incubated with NGF 100 ng/mL for 6 days; SFM supplemented with D609 15 µg/mL for 4 days; SFM supplemented with bFGF 10 ng/mL, forskolin 50 µM, SHH 250 ng/mL and 0.5 µM RA for 7 days	All three protocols led to the differentiation of DP-MSCs towards a neuronal-like phenotype, as shown by the upregulation of ChAT, MAP2, HB9, ISL1 and BETA-3	[87]
hUCB-MSCs	SFM supplemented with EGF 20 ng/mL and FGF 20 ng/mL	increase of Nestin and SOX2 Downregulation of Notch target genes, HES1 and HES5, following addition of DAPT	[88]
hPDL-MSCs	Serum-free neurobasal medium supplemented with EGF 20 ng/mL and bFGF 20 ng/mL; additionally, the cells were induced with RA for 7 days	increase of Sox2 and β3-tubulin increase of Hes-1 and Hey-1	[89]
hOE-MSCs	SFM supplemented with B27, bFGF 20 ng/mL and EGF 20 ng/mL; additionally, cells were incubated for 6 days in neurobasal medium supplemented with bFGF, FGF8b, ascorbic acid and SHH	increase of motor neuron-like markers, including Islet-1, ChAT and HB9	[90]

MSCs: mesenchymal stromal cells; AD-MSCs: adipose-derived MSCs; SFM: serum-free medium; P-PRP: pure platelet-rich plasma; A-PRF: advanced platelet-rich fibrin; CSF: cerebrospinal fluid; CM: conditioned medium; NSE: neuron-specific enolase; GFAP: glial fibrillary acidic protein; EGF: epidermal growth factor; bFGF: basic fibroblast growth factor; BM-MSCs: bone-marrow-derived MSCs; DMSO: dimethyl sulfoxide; DP-MSCs: dental pulp-derived MSCs; FBS: fetal bovine serum; BDNF: brain-derived neurotrophic; NT-3: neurotrophin-3; DCX: doublecortin; NeuN: neuronal nuclei; p75NTR: p75 neurotrophin receptor; ADH: adherent; non-ADH: non-adherent; PLP1: proteolipid-protein-1; BMP-2: bone morphogenetic protein; RC2: radial glial cells; FGF: fibroblast growth factor; RA: retinoic acid; β-ME: β-mercaptoethanol; NGF: nerve growth factor; D609: tricyclodecan-9-yl-xanthogenate; SHH: sonic hedgehog; ChAT: choline acetyltransferase; MAP2: microtubule-associated protein 2; hUCB-MSCs: human umbilical cord blood-derived MSCs; DAPT: N-[N-(3,5-difluorophenacetyl)-l-alanyl]-S-phenylglycinet-butyl ester; hPDL-MSCs: human periodontal ligament-derived MSCs; hOE-MSCs: human olfactory ecto-MSCs.

**Table 6:** Synthesis of the in vitro studies that demonstrated the effects on MSCs cultivated in serum-free medium supplemented with chemical agents used alone or combined with grown factors or other culture methods.

MSCs	Culture/Differentiation Conditions	Results	References
Human BM-MSCs	BM-MSCs exposed to bFGF 10 ng/mL, EGF 10 ng/mL and PDGF 10 ng/mL and cultivated under a serum/feeder cell-free condition	increase in neural morphology and neuronal markers including neurofilament-M, β-3 tubulin and NSE	[91]
Human BM-MSCs	SFM and exposed to EGF 20 ng/mL and RA 1 µM SFM supplemented with NT-3 10 ng/mL, BDNF 10 ng/mL, RA 0.5 µM and SHH 400 ng/mL	increase in hair-cell-like cells increase of auditory neuron-like cells	[92]
Human BM-MSCs	SFM containing grown factors; moreover, the medium was replaced and supplemented with cAMP, IBMX and ascorbic acid	Differentiation towards a dopaminergic neuronal phenotype increase of TH and Nurr 1 decrease GABAergic marker	[93]
Human BM-MSCs	Serum-free conditions and laminin-1 20 μg/mL	increase in survival and cell differentiation increase of neurites and cell bodies	[94]
Human BM-MSCs	Low-serum medium supplemented with TBA 200 nM, forskolin 50 μM and IBMX 250 μM	increase in neuronal differentiation increase of NSE, GFAP, β3-tubulin and neurofilament decrease of fibronectin	[95]
hO-MSCs	Serum-free culture media supplemented with growth factors; neural differentiation induced by SFM supplemented with N2, RA 1 µM and forskolin 5 µM	increase in neuronal and astroglial differentiation	[96]
Human fMSCs	MSCs cultivated on scaffolds consisting of polysaccharide nanofibers in SFM supplemented with SHH 200 ng/mL and RA 1 µM	increase of motor neuron-like markers, including Olig2, HB9 and ChAT	[97]
BM-MSCs from adult rats	SFM supplemented with N2 and B27; SFM and β-ME 5 mM; cocultivation with murine astrocytes	Neuronal differentiation induced by all three culture protocols Glial differentiation, when BM-MSCs were cocultivated with astrocyte, as highlighted by NeuN and GFAP levels	[98]
BM-MSCs from adult SD rats	To induce neuronal differentiation, BM-MSCs were cultivated with serum-enriched DMEM and β-ME 1 mM for 24 h; moreover, medium was replaced with serum-free DMEM supplemented with β-ME 10 mM for 3 h To induce MSCs towards cardiomyocytes, BM-MSCs were cultivated with 5-azacytidine 1 μM and zebularine 1 μM for 24 h	increase of Nestin, MAP2, Tau, Nefl, axon-like and dendrite-like processes induced by serum-free conditions and β-ME increase of troponin-T, GATA-4 and Nkx2.5 induced by 5-azacytidine and zebularine	[99]
BM-MSCs isolated from female Fisher rats	Serum-free medium supplemented with forskolin 10 μM and Serum-free medium supplemented with ascorbic acid 2 phosphate 50 μg/mL	Forskolin induced a transient neural-like morphology, as shown by the presence of neurite-like processes increase of NSE, neurofilament 200 and β-3 tubulin increase in cell survival and viability induced by ascorbic acid 2 phosphate and serum-free conditions	[100]
AD-MSCs from C57BL/6 mice	Medium containing a synthetic serum KOSR used alone or supplemented with β-ME 0.1 or 1 mM	increase both of neural differentiation and neuronal markers Pax6, nestin and MAP2 induced by KOSR decrease of neural differentiation induced by β-ME integration	[101]

MSCs: mesenchymal stromal cells; BM-MSCs: bone-marrow-derived MSCs; bFGF: basic fibroblast growth factor; EGF: epidermal growth factor; PDGF: platelet-derived growth factor; NSE: neuron-specific enolase; SFM: serum-free medium; RA: retinoic acid; NT-3: neurotrophin-3; BDNF: brain-derived neurotrophic factor; cAMP: cyclic adenosine monophosphate; IBMX: 3-isobutyl-1-methylxanthine; TH: tyrosine hydroxylase; GABA: gamma-aminobutyric acid; TBA: 4β-12-O-tetradecanoylphorbol 13-acetate; GFAP: glial fibrillary acidic protein; hO-MSCs: human olfactory mucosa-derived MSCs; fMSCs: first-trimester MSCs; SHH: sonic hedgehog; Olig2: oligodendrocyte lineage transcription factor 2; ChAT: choline acetyltransferase; β-ME: β-mercaptoethanol; NeuN: neuronal nuclei; SD: Sprague Dawley; MAP2: microtubule-associated protein 2; Nkx2.5: NK2-transcription-factor-related locus 5; AD-MSCs: adipose-derived MSCs; KOSR: knockout serum replacement.

## Data Availability

No new data were created or analyzed in this study. Data sharing is not applicable to this article.
